# Effect of vitamin C and vitamin E on lung contusion: A randomized clinical trial study

**DOI:** 10.1016/j.amsu.2018.10.026

**Published:** 2018-11-09

**Authors:** Davoodabadi Abdoulhossein, Iman Taheri, Mohammad ali Saba, Hossein Akbari, Shima Shafagh, Asemi Zataollah

**Affiliations:** a General and Thoracic Surgeon Associated Prof Kashan University of Medical Sciences, Departments of General Surgery Trauma Research Center, Iran; b Kashan University of Medical Sciences, Departments of Surgery, Iran; c Kashan University of Medical Sciences, Departments of Pulmonology, Iran; d Department of Biostatistics and Epidemiology, Social Determinants of Health (SDH) Research Center, Kashan University of Medical Sciences, Kashan, Iran; e Kashan University of Medical Sciences, Departments of General Surgery Trauma Research Center, Iran; f Kashan University of Medical Sciences, Departments of Nutrition, Iran

**Keywords:** Lung contusion, Chest trauma, Ascorbic acid, Vitamin E, Lung contusion, LC, Arterial blood gas, ABG, C-reactive protein, CRP

## Abstract

There is association between lung contusion (lC) and a progressive inflammatory response. The protective effect of vitamin C and vitamin E, as strong free radical scavengers on favourite outcome of (LC) in animal models, has been confirmed.

**Design:**

to evaluate the effect of vitamins, E and C on arterial blood gas (ABG) and ICU stay, in (LC), with injury severity score (ISS) 18 ± 2, due to blunt chest trauma.

**Methods:**

This study was a randomized, double-blind, placebo-controlled clinical trial. Patients with (ISS) 18 ± 2 blunt chest trauma, who meet criteria, participated in the study. A total of 80 patients from Feb 2015 to Jun2018and were randomly divided into 4 groups. Patients received intravenous vitamin E (1000IU mg), was (group I); intravenous vitamin C (500) (group II). Vitamin C + vitamin E = (group III), and intravenous distilled water = (control group) or (group IV). ABG, serum cortisol, and CRP levels were determined at baseline, 24 h and 48 h after the intervention.

**Results:**

a significant decrease in ICU stay in group III compared to other groups (p < 0.001). Co-administration of vitamin C and vitamin E showed significant increases pH (values to reference range from acidemia”), oxygen pressure, and oxygen saturation in group III compared to other groups (p < 0.001). A significant decrease in carbon dioxide pressure was also detected after receiving vitamin C and vitamin E in group III, compared to other groups (p < 0.001). There was no significant difference cortisol and CRP levels between groups after the intervention.

**Conclusion:**

Co-administration of vitamin C and vitamin E, improve the ABG parameters and reduce ICU stay.

## Highlights

• Most previous studies about pulmonary contusion were performed on animal model, this is a human study in surgery ICU.
• Administration of vitamin C and vitamin E showed protective effects on pulmonary contusion, co-administration of them also was more effective.
• PH values, oxygen pressure, and oxygen saturation was significantly increase in group III compared to other groups (p < 0.001). carbon dioxide pressure also was significantly decrease in group III.
• Mean ICU stay in group III (co-administration of vit C + E compare with control group, significantly decreased, p < 0.001).

## 1 Introduction

Lung contusion (LC), is the most frequent intrathoracic injury due to blunt chest trauma, despite improvement in treatment modalities, still has up to 20% mortality rate in severely thoracic trauma [1].The majority of (LC) patients have multiple injuries with thoracic trauma, while 10–20% of cases are affected in an isolated manner [2,3]. Lung contusion (LC) is considered as a crucial risk factor for the development of pneumonia, acute lung injury and acute respiratory distress syndrome [1]. Lung contusion can promote systemic inflammatory activation and consequently leads acute respiratory failure due to alveolar collapse and impaired fluid clearance [4].Although the exact mechanisms of LC are not yet understood, and many efforts have been made to describing pathophysiological changes after blunt trauma, however, the association between LC and a progressive inflammatory response which mediated by local and systemic immunological alteration, has been well described [5,6]. In LC, alveolo-capillary membrane permeability is increased as a result of cytokines, proteolytic enzymes, and reactive oxygen species, are released from macrophages [7]). Standard treatment options of LC, is Supportive management techniques including supplemental oxygen cardiopulmonary monitoring and there are limited treatment options available for this condition, however controlling the activation of a secondary inflammatory response is great importance for moderate to severe patients with LC [5,6]. Vitamin C and vitamin E are proposed as strong free radical scavengers [8].The protective effect of vitamin C and vitamin E on the biochemical and histopathological outcome of LC in animal model of blunt trauma has been recently confirmed [9], Vitamin C and vitamin E could be easy to administer in lung contusion. We hypothesize that vitamin C and vitamin E or combination of them, may have potential Protective effects on LC recovery and may improve the pathophysiological changes, arterial blood gas (ABG) parameters and decrease ICU stay, serum cortisol and CRP in LC due serious to severe blunt chest trauma.

## 2 Patients and methodology

### 2.1 Study design

This study was a randomized, double-blind, placebo-controlled clinical trial, in Patients with series to severe blunt chest trauma, who meet the inclusion criteria and gave informed consent, to participate in the study.

This study, conducted in Trauma Research Center, Level 3 Trauma Center registry, on patients admitted in ICU surgery of complex teaching hospital, between Feb 2015 to Jun 2018 to assess the Protective Effect of Vitamin C and vitamin E on Arterial Blood Gas parameter and ICU length stay on patients, with diagnosis of pulmonary contusion due to blunt trauma.

The study was approved by the University of Medical Sciences Clinical Research review board, Ethics Committee and registered as Identifier: IR.KAUMS.REC.1395.153 and also registered in Iranian Registry of Clinical Trial (IRCT) with reference number of IRCT20180205038627N1.

Diagnosis of lung contusion: was made by clinically manifestation of respiratory distress, hypoxemia, tachypnea, diminished breath sounds, hypercarbia, chest wall pain and subcutaneous emphysema, at the time of admission. 1-3rib fractures and flail chest, mild to moderate hemothorax, also was detected on plain radiography.

The feature of lung Contusion on CT scan as a final diagnostic tool was focal, non-segmental areas of lung parenchyma opacity, Laceration with Simple pneumothorax, mild to moderate air leak and Nonexpanding hemothorax which stopped after chest tube insertion up to 72 h.

Injured lung was mostly peripherally near rib fractures area which apparent during24 h after blunt trauma. The involved Lung contusion, was mostly unilateral, one lobe, and rarely both lobe.

All patients who gave informed consent were enrolled in the study according to eligibility criteria, listed as follows.

### 2.2 Inclusion criteria

Unilateral chest injury, being in serious to severer traumatic condition, although we tried to select the injured patients to be more isolated however in 32% patients, had minor associated injuries as follow:

Minor ECG abnormality: nonspecific ST changes, transient sinus tachycardia, intraabdominal trauma such as mild nonexpanding Subcapsular Haematoma of spleen and liver, gross haematuria, with normal sonography or nonexpanding perirenal haematoma without urinary extravasation, minor limb injures treated with skin traction or long cast. So our patients on base of Abbreviated Injury Scale(AIS.) for head and neck, face, thorax, abdomen/pelvis extremity and external injury; which describes the severity of injury to one body region on a six-point scale: 1 minor, 2 moderate, 3 serious, 4 severe, 5 critical, 6 un survival and Organ Injury Scaling for multiple injuries was Grade1and 2 [10]) Therefore injures severity score estimated between 18±2- [11]).

### 2.3 Exclusion criteria

Penetrative trauma, patients in critical condition, complicated patients, needing intubation more than 3 days, requiring emergently major surgical intervention such as brain, abdominal and major orthopedic surgery (pelvic, femur fracture), patients with opium addiction, chronic obstructive pulmonary disease, asthma Glasgow Coma Scale (GCS), less than 13, major associated trauma, pulmonary edema, blood transfusion and thromboembolic effects in long bone fracture, were excluded to minimize confounding the cause of adverse events, any other potential confounder of pure study of lung contusion such as pneumonia, adult respiratory distress syndrome also was excluded, Combined chest wall Injuries with lung contusion were: fracture rib1-3, Laceration of Scapular body fracture of clavicle, sternum, Unilateral flail segment <4 ribs, hemothorax, which drained with chest tube.

### 2.4 Sample size calculation

In order to assess the Protective Effect of vitamin E, C and E + C, on LC, with confidence interval 95%, power 80% and effect size of 0.4, with use of Cohen table, 18 recruitments for each group were needed with an estimated a drop-out rate of 10%, a sample size of 20 patients in each group were chosen. Recruitment protocol integrity for this study, with considering 20 recruitments for, each group, would be completed within 2 years.

### 2.5 Randomization

Randomization assignment by permuted blocked method was conducted with using computer-generated random numbers. Randomization and allocation concealment were conducted by the researchers and participants and were carried out by a trained staff at the clinic. Another person, who was not involved in the trial and not aware of random sequences, assigned the subjects for taking supplements. Patients’ diagnosed LC who meets the inclusion criteria were randomized to vitamin E (group I); vitamin C = (group II), vitamin C + vitamin E = (group III), and control group or (placebo (group IV). The eligibility of patients was obtained on base of Injury Severity Score (ISS), between 18±2and nearly all groups with, acceptable homogeneity and similarity, were randomized to four groups.

### 2.6 Pere-intervention considerations

Each group received the current standard supportive care: Cleared tracheal secretions, enough fluids administered without exacerbating edema (euvolaemia), hemoglobin was above 11 mg/dl,electrolytes were in normal range, There were no differences in physiotherapy, appropriate analgesia and subcutaneous heparin. Adequate oxygen supplementation offered between each groups. Systolic blood pressure in the field of ICU was, above mean110mmhg. Heart rate, mean 85/min Respiratory rate, mean19/min, mean oxygen saturation 91%and po2,81mmhg hence, all groups was thermodynamically stabilized.

Before doing intervention we tried all groups to being physiologically homogeny as much as possible. Patients if has been intubated during first72 h of accident, and have possible measure for extubation, were extubated. All critical care treatment decisions including tracheal intubation, mechanical ventilation if need, extubation, length of ICU staying with prospect of intention to treatment, under the direction of thoracic surgeon and anesthesiologist, who was not aware from protocol, was done.To prevent atelectasis and lung infection we did pain-control with pharmacologic therapy, rather than intercostal nerve black or catheters insertion and obliged posture at, at least, 45°, interventions started after extubation and patients adequately were prepared for Intervention.

ICU length stay also was measured after extubation, ICU stay may more precise than hospital stay, although majority of patients after transfer to ward discharged 2-day latter, if had no any combined injuries that required managed by other surgeon (continue skin traction brain problem).

### 2.7 Protocol

Every patient, who met the inclusion criteria, was randomly divided to 4 subgroups. Group I (n = 20) received intravenous infusion vitamin E (1000 IU in 50 ml of emulsion). In patients of group II (n = 20) intravenous infusion vitamin C (500 mg in50 ml normal saline) was administered; while in individuals of group III (n = 20) vitamin C (500 mg) + vitamin E (1000 IU) was administered. Patients in group IV (n = 20) were considered as control group and received intravenous infusion distilled water (5 mLin50 ml normal saline), By pump, steady, during up to 30minets.

The trial process was done during 24–48 h of ICU admission. Arterial blood in air-free heparinized syringe was prepared, labeled with the patient's full name, chart number, date and time of collection and immediately delivered to ICU Blood gas analysis sampling kits in our ICU ward.

At the same time, from each groups of patients also 5 mL of venous blood was drawn as a base, 24 h, and48 h latter and sent to laboratory of hospital at 8 a.m. for measuring quantify serum cortisol (as a parameter of better predict treatment response and CRP levels as high levels indicating systemic inflammation. (12).

After 48 h, the anti-oxidants administration, was stopped and patients monitored by clinical presentation and pulse oximetri.

### 2.8 Data collection

Data collection was commenced in Kashan Trauma Research Center after approval study. During the study period, 1580 consecutive patients were admitted to in our center with chest trauma. Penetrative injuries 83cases (19%) complicated, crucial patients were not studied, patients that requiring prolonged intubation, major associated injury ISS more than 21 also excluded, 80patients were screened for inclusion into this study, age; sex; mechanism of injury; Glasgow Coma Scale score mortality, morbidity, vital signs including, SBP, heart rate, and respiratory rate presence of other injury as well as the ISS, AIS score for the head/neck, face, thorax, abdomen/pelvis, extremity, and external injury.

ABG parameter, before, after 24 h, and48 h, data were prospectively collected for each groups participating in the study.

### 2.9 Statistical analysis

Data were analyzed using IBM SPSS v21and presented as mean ± standard deviation. To compare treatment groups, Anova and Tukey's HSD test for quantitative data, chi square and fisher's exact test for qualitative data were used. Finally, analysis of variance with repeated measurement for investigating of effect of time and interaction of time and treated group for assessing and comparing of groups was applied. P value < 0.05 was considered to be significant.

### 2.10 Post-intervention considerations

Standard monitoring of groups, (closed clinical observation and pulse oximetri), continuing physiotherapy, appropriate analgesia and anticoagulant, adequate oxygen supplementation and liquid diet offered in each groups until the patients transferred to ward.

## 3 Results

Out of 80 patients, 54 (67.5% were males and the rest were female. Male to female ratio was 2:1, In group1to iv, 65%,70%,65%, and 70% were male respectively, the mean age was 40.06 ± 8.96 years.82% of cases were due to motor vehicle accident and the rest due to falling. (Table 1). Also GCS of patients in 4 groups 25%, 65%, 65%, and 70% was 15. Statistical analysis showed no significant difference among groups in terms of age, (P-value) = 0.99, sex, (P-value = 0.854) and GCS score. Table 1. Mean ICU stay after intervention in group I = 5.2 ± 1.74 m, group II = 5.5 ± 1.73, group III = 3.5 ± 0.5.and control was 5.2 ± 1.67 days, (P-value<0.001) (Table 1). The mean ICU stay periods in control and group III was (5.2 ± 1.67and 3.5 ± 0.5 days, p < 0.001); Table 1 mean ICU stay periods was not found to be Significant difference among other groups (P-value<0.98), (Table 1). Turkey's Post hoc test showed significant difference between group III with control(P > 0.5), but there is no difference between group I and control (P-value<0.5).and also no significant difference between parameters of pH، PO2، PCO2، O2sat, Cortisol, CRP in the beginning of study in four groups (p = 0.03). (Normal Values and Acceptable Ranges of the ABG Elements: Oxygen Saturation (93–100%) pH = 7.4 (7.35–7.45). pCO2 = 40 mmHg (35–45 mmHg). pO2 = 90 mmHg (80–100) mmHg.) (Normal Values of Morning serum cortisol (8am) 140–690 nmol/L Quantitative C-Reactive Protein (CRP) = 0.0–4.9 mg/L)

**Table 1:** Demographic characteristics, GCS and ICU stay in lung contusion patients.

groups		Vit E	Vit C	Vit C + E	Control	P.value
Sex	Male	13(65%)	14(70%)	15(75%)	12(60%)	0.854^a^
	Female	7(35%)	6(30%)	5(25%)	8(40%)	
GCS	13	1(5%)	1(5%)	1(5%)	1(5%)	0.982^b^
	14	8(40%)	6(30%)	6(30%)	5(25%)	
	15	11(25%)	13(65%)	13(65%)	14(70%)	
Age(mean ± SD)		39.9 ± 9.3	40.35 ± 8.94	40.05 ± 9.51	39.95 ± 9.05	0.999^c^
ICU stay after intervention		5.2 ± 1.74	5.5 ± 1.73	3.5 ± 0.5	5.2 ± 1.67	0.001^c^

a Chi square test.

b Fisher's exact test.

c One way Anova.

Before the intervention, pH (values to reference range from academia)” were not significantly different between groups and after 24 h and 48 h were not significantly improved except group III (Table 2).

**Table 2:** Alternation in ABG parameters before and after the intervention in different groups.

groups		Group E	Group C	Group E + C	control	P1	P2
pH	before	7.25 ± 0.06	7.24 ± 0.044	7.25 ± 0.057	7.25 ± 0.051	<0.001	<0.001
	After24 h	7.33 ± 0.033	7.33 ± 0.031	7.35 ± 0.005	7.31 ± 0.031		
	after 48 h	7.33 ± 0.031	7.34 ± 0.03	7.39 ± 0.011	7.32 ± 0.034		
PO2	before	81.7 ± 4.64	82.85 ± 3.25	82.5 ± 4.67	80.85 ± 2.9	<0.001	<0.001
	After24ho	86.7 ± 4.64	87.85 ± 3.29	90.5 ± 4.67	84.85 ± 2.9		
	after 48 h	91.7 ± 4.64	92.85 ± 3.29	99.5 ± 4.67	88.85 ± 2.9		
PCO2	before	41.7 ± 1.71	42.15 ± 1.81	42.45 ± 1.66	42.15 ± 1.53	<0.001	<0.001
	After24ho	38.7 ± 1.71	39.15 ± 1.81	37.45 ± 1.66	39.15 ± 1.53		
	after 48 h	37.7 ± 1.71	38.15 ± 1.81	34.95 ± 0.82	39.15 ± 1.53		
O2Sat	before	92.15 ± 1.49	92.25 ± 1.55	92.05 ± 1.27	91.95 ± 1.35	<0.001	<0.001
	After24ho	97.15 ± 1.49	97.25 ± 1.55	98.05 ± 1.27	95.95 ± 1.35		
	after 48 h	97.15 ± 1.49	97.25 ± 1.55	98.45 ± 0.82	95.95 ± 1.35		

Before the intervention, the results of ABG parameters were not significantly different between groups. Accordingly, pH, PO2, PCO2, and O2 saturation were similar in different groups with no significant difference (p = 0.85, p = 0.39, p = 0.57, and p = 0.92, respectively). After starting the intervention, the increase in pH, PO2, and O2 saturation was significantly higher in group III compared to other groups (p < 0.001). The decrease in PCO2 was also significantly higher than other group (p < 0.001). Table 3 alternation of, serum cortisol and CRP.

**Table 3:** Depicts the alternation of serum cortisol and CRP before and after the intervention in different groups.

Intervention		group1(vitE)	Group2(vitC)	Group3(vitC)	Control	P1	P2
Serum Cortisol(nmol/L)	before	228.5 ± 61.3	258.4 ± 56.7	242.25 ± 58.4	245 ± 65.8	N.S	N.S
	after24 h	207.5 ± 61.3	237.4 ± 56.7	222.25 ± 58.4	225 ± 65.8		
	After48 h	182.5 ± 61.5	212 ± 56.7	197.2 ± 58.4	201 ± 65.8		
Serum CRP	before	2.17 ± 5.7	1.83 ± 5.7	1.97 ± 6.3	2.31 ± 3.8	0.001	0.102
	after24 h	26.2 ± 5.2	1.61 ± 5.2	58 ± 5.5	1.96 ± 5.4		
	after48 h	2.1 ± 3.55	84.1 ± 3.7	1.94 ± 1.4	2.31 ± 8.3		

P1 Effect of time P2 Effect of interaction time and group.

Morning serum cortisol (am) 140–690 nmol/L Quantitative C - reactive protein (CRP) = 0.0–4.9 mg/L.

Serum cortisol level, after the intervention, decreased values, but was not significant among groups before the intervention (p = 0.247) table The serum concentration of CRP as inflammatory biomarker measured, also after the intervention, decreased moderately, but was not significantly. (P-value = 0.102) Table 3.

### 3.1 Outcome

Since we selected the patents in fairly good general condition, the course of disease was well, all of patients were discharged from hospital with good condition and 30 days’ follow-up was favourable without any morbidity or mortality.

**Fig. 1:**
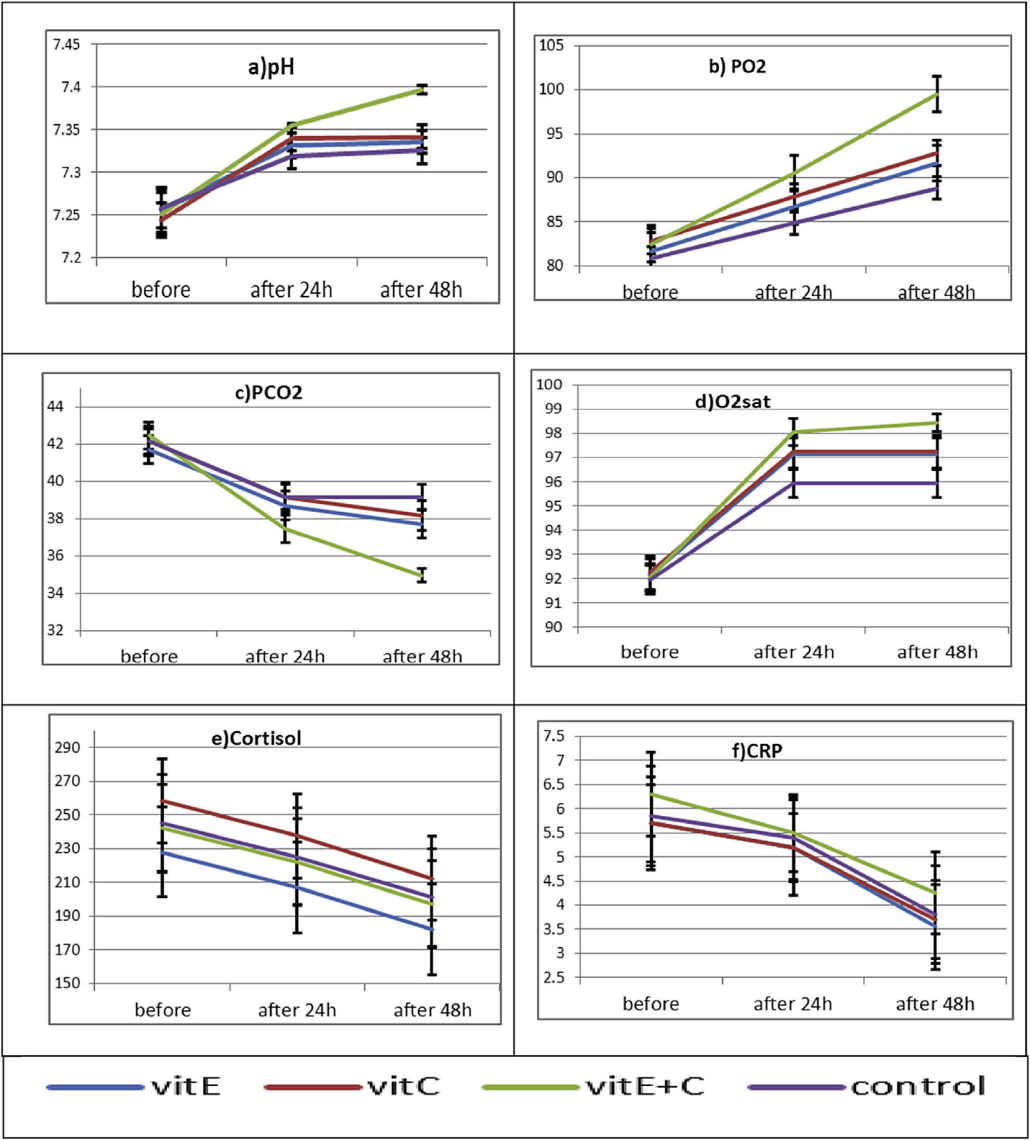
ABG parameters improvement with Vit E,C administration.

Fig. 1 depicts the alternation in ABG parameters before and after the intervention in different groups.

## 4 Discussion

Several animal trials have been conducted to understand, the inflammatory mechanisms and the potential effect of anti-inflammatory drugs on treatment of LC after blunt thoracic trauma [[13], [14], [15], [16]]).and the protective effect of vitamin C and vitamin E on the biochemical and histopathological improvement of LC in animal model has been confirmed [9,[13], [14], [15],^17^,^18^], in a rat model of LC, administration of vitamins C and E improved both the PaO2 and PaCO2 levels. [15], but there is little published human study that assess the inflammatory mechanisms and the potential protective effect of anti-inflammatory of vitamin E and C, on improving ABG parameter and length of ICU stay. Possible reasons are many heterogeneity in chest trauma, complexity, variability of patients and potential confounders of pure study are considerable, isolated cases are relative rare anatomic and physiologic differences that exist between patients, leads some difficulty in human study.

Our study revealed administration of vitamins C and E has protective effect, on ABG parameter and length of ICU stay Possible reasons for this beneficial results, is due to the inflammatory mechanisms and the potential effect of anti-inflammatory vitamin E and C supplementation specially when administered together. Lung contusion in both human and animal models is characterized by an intense inflammatory response in the pulmonary parenchyma. Local and systemic immunological alterations are known as the important factors of progressive inflammatory response and systemic inflammatory activation, may leads acute respiratory failure [4,15].

In lung contusion, components of blood enter the tissue, macrophages, neutrophils and leukocyte-mediated secondary inflammatory response inflammatory cells lead to inflammation, increased alveolocapillary permeability and pulmonary edema, which leads, hypoxemia, hypercarbia, and increased work of breathing [[19], [20], [21]]),inflammation may also affect the contralateral lung [7] and If this inflammation became severe can lead acute respiratory distress syndrome [7].The water content of the lung increases over the first 72 h after injury, potentially leading to frank pulmonary edema in more serious cases [19]. so every effort may be effective earlier than this period. The role of neutrophil influx in development of PC is also well established [23]. A sudden increase in pro-inflammatory mediators has been also detected in alveolar level as a result of macrophages and neutrophils activation [24]). Reactive oxygen species can be subsequently produced from alveolar macrophages [15])These findings suggest the potential role of oxidants and antioxidants in the mechanism of lung contusion.

Although the standard treatment options of LC are Supportive management techniques, supplemental oxygen and cardiopulmonary monitoring, however vitamin C and vitamin E were applied, improved the ABG perimeters, co-administration of vitamin C and vitamin E have more beneficial than alone, in decreasing the ICU stay, improving the pH acidemia values, oxygen pressure, and oxygen saturation, and decreasing the carbon dioxide pressure. Co-administration of vitamin C and vitamin E also have decreased, serum cortisol levels, which used as parameter to judge possible treatment response 12), at same time measured during study parallel to improving the ABG results, decreased moderately, However it was not significant.(“(p > 0.05)"), and C-reactive protein (CRP) was high levels, indicating systemic inflammation and exponential fall after antioxidants administration at baseline, 24 h and 48 h were measured synchronously with ABG also showed decreased moderately, but was not significant (P > 0.05).

Vitamin E and C administration have been showed protective properties, in other organs, Vitamin C decrease the length of hospital stay, drainage volume in the ICU and in the first 24 postoperative hours, intubation time in patients after cardiac surgery [25]).

Administration of vitamin E reduces acute inflammatory cell influx, and suppresses collagen formation in lung tissue; vitamin E could be used in combination with corticosteroids for protection against chemical-induced lung injury [26]). The antioxidative activity of vitamin E was demonstrated in the study of Cristante et al., in a rat model of spinal cord injury they suggested that rats with spinal cord injury may benefit from vitamin C administration to reduce the inflammatory response [27]. Acute inflammatory cell influx and collagen formation is also suppress after chemically induced lung injury by the administration of vitamin E [28]).Oxygen-derived free radicals are omitted by Vitamin C as a well-known scavenger of these radicals [29]) The results of our clinical trial were in accordance with the previously animal study articles and confirmed the protective effects and anti-inflammatory role of vitamin E and vitamin C in patients with LC due to blunt chest trauma, particularly, had best outcome if these two supplements are administered concomitantly.

## 5 Conclusion

Our results suggest that administration of vitamin C and vitamin E have favourable effects on pulmonary contusion management, co-administration of vitamin C and vitamin E is more effective and continue during whole ICU stay. Additional studies with large patient sample size are required to confirm these findings.

### 5.1 Strengths and limitations of the study

This study carried out in Trauma Research Center in a complex University Hospital which is tertiary and well equipped with 735bed, in all fields of surgeries specialty ward located in north of Isfahan province and affiliate to Kashan medical sciences. It covers population near 500000. According to our trauma center registry, annually 6700–7000 traumatic patients are admitted and near 2300admimsions are more than24 h hospital stay. Most previous studies were performed on animal model, this study is carried out on real patients and results of such experiences less has been published. Although we tried all groups’ severity of injury, being homogen as much as possible, however there are many heterogeneity in chest trauma, isolated cases are relative rare, mostly have associated multiple injury, anatomic and physiologic differences that exist between patients, leads some bias and similarity in groups became difficult and hence ISS also not sufficient accuracy so, further research on Lc needs to be done next with large patient sample size and isolated chest trauma for better ISS accuracy, continuing co-administration of vitamin C and vitamin E more than 48 h and finally measurement of antioxidants concentration in the time of base and end of protocol for optimal effectiveness.

## Ethical approval

Research review board, Ethics Committee and registered as Identifier: IR.KAUMS.REC.1395.153.

## Sources of funding

This study was thesis of ImanTaheri(general surgery residency) under supervision of davoodabadiabdoulhossein conducted in Kashan University of Medical Sciences. This project was funded by Kashan University of Medical Sciences.

## Conflicts of interest

The authors declare no conflict of interest.

## Research registration number

Iranian Registry of Clinical Trial (IRCT) with reference number of IRCT20180205038627N1.

## Guarantor

Abdolhossein Davoodabadi is the Guarantor of this study and accept full responsibility for the work and/or the conduct of the study, have access to the data, and controlled the decision to publish.

## Provenance and peer review

Not commissioned, externally peer reviewed.
